# To screen or not to screen: complexity of *SDHA* mutation management

**DOI:** 10.1530/EO-25-0048

**Published:** 2026-01-29

**Authors:** Einas Mohamed, Rosalind Eeles, Terri McVeigh, Daniel Morganstein

**Affiliations:** ^1^ Imperial College Healthcare NHS Trust, London, UK; ^2^ The Royal Marsden NHS Foundation Trust, London, UK; ^3^ The Institute of Cancer Research, London, UK; ^4^ The Royal Marsden NHS Foundation Trust, Thyroid Unit, London, UK; ^5^ Chelsea and Westminster Hospital, NHS Foundation Trust, Endocrinology Department, London, UK

**Keywords:** SDHA, variants, germline, GISTs, surveillance

## Abstract

**Summary:**

Pathogenic germline variants in the genes encoding the various subunits of the succinate dehydrogenase (SDH) enzyme complex are strongly associated with hereditary phaeochromocytomas and paragangliomas (PPGLs). Germline pathogenic variants (PGVs) in the SDHA gene are also found in individuals with wild-type gastrointestinal stromal tumours (GISTs) and paragangliomas. However, the penetrance of SDHA variants is relatively low. Only a minority of carriers will develop tumours as a result of the genetic condition. As a result, careful clinical interpretation is therefore required to avoid unnecessary over-investigation or undue concern. Current national guidelines recommend that SDHA variants should only be considered actionable (that is, to lead to surveillance in the proband and family screening) if they are identified in individuals with a personal or family history of an SDHA-related tumour. Nevertheless, SDHA variants are increasingly detected as a result of whole genome sequencing or multigene panel testing. This emphasises the importance of clinical context when deciding on surveillance or family testing. We describe three carriers of germline *SDHA* variants. The first case is a 72-year-old male with a history of prostate cancer and GIST, where genetic testing revealed germline variants in BRCA2 and SDHA. Tumour-derived DNA from his GIST demonstrated a somatic PDGFRA driver mutation, and SDH staining was preserved, indicating a sporadic PDGFRA-driven GIST rather than a classical SDHA-deficient tumour. While truncating SDHA variants such as this one have been reported in SDH-deficient GIST, the current UK practice considers them low penetrance and typically not actionable in the absence of SDH-deficient pathology. Accordingly, cascade testing of the SDHA variant was not recommended in his family, and no surveillance for SDH-associated tumours was initiated, although we recognise that some international guidelines may take a more precautionary approach. The second case features a 59-year-old female with wild-type GIST and a family history of brain tumours and breast cancer. Testing of tumour-derived DNA identified an *SDHA* variant, later confirmed of germline origin. Surveillance for other SDH-associated tumours for the proband and cascade testing for her relatives were recommended. The third case involves a man in his sixties with prostate cancer, found to carry an incidental SDHA variant after undergoing broad cancer predisposition panel testing. He had no personal/family history of SDH-associated tumours, and his prostate cancer showed strong *SDHA* expression, indicating that the variant was non-actionable, and no surveillance for SDH-associated tumours or familial cascade testing was recommended. These cases underscore the importance of interpreting *SDHA* variants carefully, as the identification of germline *SDHA* variants does not always indicate the need for aggressive surveillance or intervention.

**Learning points:**

- Germline *SDHA* variants increase the risk of PPGLs and wild-type GISTs.
- The penetrance of germline *SDHA* variants in carriers without an *SDHA*-related tumour is low.
- Careful clinical assessment is essential to determine if a patient with an *SDHA* germline mutation has an *SDHA*-related tumour.
- This is particularly complex in GISTs, as only wild-type GISTs (those without somatic variants in c-Kit or PDGFRA) are likely to be *SDHA*-related.
- Immunohistochemistry for expression of SDH subunits can help clarify whether a tumour is related to *SDHA* variants.

## Background

Phaeochromocytomas (PCCs) and paragangliomas (PGLs) are rare neuroendocrine tumours originating from chromaffin cells. These tumours are known for their strong hereditary basis, with germline mutations identified in around 30% of cases – making them among the most genetically driven of all neoplasms (1).

Over the past few decades, several PCC/PGL predisposition genes have been identified, including the genes encoding for subunits of the succinate dehydrogenase (SDH) enzyme complex (2, 3). SDH plays a critical role in both the tricarboxylic acid cycle and the mitochondrial respiratory chain, contributing to oxidative metabolism and electron transfer. Variants in the genes encoding SDH subunits have been shown to predispose individuals to PCC/PGL, indicating a need for regular surveillance in affected carriers (4).

In recent years, variants in the *SDHA* subunit have garnered attention not only for their association with PCC/PGL but also for their role in the development of gastrointestinal stromal tumours (GISTs) (5). However, the penetrance of *SDHA* variants is significantly lower compared to variants in the other three SDH subunit genes, with studies suggesting that only around 10% of carriers will develop a related tumour by age 70 (6). This low penetrance has led to the development of updated clinical guidelines, which recommend that surveillance and family testing for *SDHA* variants should only be considered where the carrier has presented with an *SDHA*-related tumour (7).

The increasing use of genetic testing, particularly through multigene panels, has led to an increase in the identification of *SDHA* variants in individuals without a clear history of *SDHA*-related tumours. This presents a clinical challenge, as it requires careful decision-making to ensure that ongoing surveillance and family testing are performed only when likely to be beneficial, minimising the burden on individuals and their families. These developments highlight the need for a nuanced approach to genetic counselling and clinical management in patients with *SDHA* variants, particularly those who do not have a clear clinical indication for further investigation.

As more *SDHA* variants are identified through genetic testing, particularly in patients without a personal or family history of PCC/PGL, it is increasingly important to implement current guidelines for when further surveillance and family testing are warranted.

In this context, we present three cases where *SDHA* variants were identified in individuals who did not present with a paraganglioma or phaeochromocytoma. These cases highlight the complexities of clinical assessment in the era of multigene panel testing, and we propose an algorithm to guide decision-making in similar situations.

## Case 1

A 72-year-old male with type 2 diabetes was diagnosed with a gastrointestinal stromal tumour (GIST) in 2019, managed with partial gastrectomy. Shortly thereafter, a raised prostate-specific antigen (PSA) on precautionary testing led to a diagnosis of prostate cancer, treated initially with total prostatectomy. Low residual PSA post-surgery prompted consideration of salvage radiotherapy; however, before this was commenced, he underwent colonoscopy for constipation, during which two small polyps (inflammatory and low-grade dysplasia (LGD)) were removed. In 2024, another colonoscopy for suspected pancreatic or radiotherapy-related issues identified a sub-centimetre LGD polyp, but no malignancy.

Given the occurrence of two primary cancers, germline testing was undertaken following genetic counselling. This identified pathogenic variants in BRCA2 (exon 14–16 deletion) and SDHA (c.91C>T, p.Arg31*). Tumour testing revealed a somatic PDGFRA driver mutation, confirming the GIST was not wild type. Immunohistochemistry (IHC) demonstrated preserved SDHA staining, suggesting that the GIST was unlikely to be SDHA-related. Plasma metadrenalines were within normal limits (metadrenaline 385 pmol/L, normetadrenaline 854 pmol/L and 3-methoxytyramine < 85 pmol/L), and there was no clinical or radiological evidence of paraganglioma.

While truncating SDHA variants have been associated with SDH-deficient GIST in the literature, in the UK clinical practice context, this variant was regarded as low penetrance and therefore non-actionable, with no further surveillance recommended. We acknowledge, however, that other health economies may adopt a different approach, given published reports of SDHA-deficient GIST arising in patients without family history.

## Case 2

A 59-year-old female was referred after an incidental finding of a GIST on a CTPA scan in July 2022. She had a family history of brain tumours and breast cancer. The GIST was confirmed by endoscopic ultrasound. Testing of tumour-derived DNA identified no variants in *KIT* or *PDGFRA* but did identify a variant in *SDHA* (*SDHA* c.1663G>T, p.Gly555Ter) at a high variant allele frequency (91.2%), later confirmed of germline origin after review in a Cancer Genetics clinic. Although negative findings for phaeochromocytomas or paragangliomas and plasma metadrenalines were within normal reference limits (NR metadrenaline < 510, normetadrenaline < 1,180 and 3-MT < 180 pmol/L), the finding of a germline *SDHA* variant in the context of a proband with an SDH-related GIST indicated the need for ongoing surveillance for other SDH-associated tumours. In addition, family genetic testing was recommended, to enable surveillance for *SDHA*-related tumours in carrier relatives.

## Case 3

A man in his 60s, with hypertension, hypercholesterolaemia and primary hypothyroidism, underwent radical prostatectomy for prostate cancer in June 2021. Subsequent germline testing (broad multigene cancer predisposition panel performed after review in a Cancer Genetics clinic) identified an SDHA c.223C>T (p.Arg75*) truncating variant.

He reported night sweats but denied palpitations, flushing or other classical symptoms of catecholamine excess. Whole-body MRI showed no evidence of paragangliomas or prostate cancer recurrence. Plasma metadrenalines were normal (metadrenaline 143.8 pmol/L (NR < 510), normetadrenaline 1,178.3 pmol/L (NR < 1,180), 3-methoxytyramine < 75 pmol/L (NR < 180)). Histology confirmed poorly differentiated adenocarcinoma, with IHC showing preserved SDHA and SDHB expression, indicating that the prostate cancer was unlikely to be SDHA-related.

Although truncating SDHA variants have been associated with SDH-deficient tumours in published series, in the UK setting, such variants are considered low penetrance and typically not actionable in the absence of SDH-deficient pathology. Accordingly, no formal surveillance or cascade testing was recommended. However, recognising variation in practice internationally, the patient was counselled that other guidelines may favour surveillance. He elected to repeat plasma metadrenalines annually alongside PSA monitoring on a private basis.

## Discussion

We describe three individuals in whom germline genetic testing, prompted by a personal history of cancers other than phaeochromocytomas and paragangliomas (PPGLs), identified SDHA variants. Two of these patients had gastrointestinal stromal tumours (GISTs), and one had prostate cancer. While truncating SDHA variants are recognised to predispose to SDH-deficient GISTs and PPGLs, clinical interpretation remains nuanced. Careful assessment is required to establish whether the index tumour is SDHA-related, as this distinction directly informs whether the germline finding should be considered actionable. This is important to avoid both missed opportunities for surveillance and unnecessary over-investigation with its associated patient and family burden.

In the first two cases, the index tumour was a GIST. GISTs are most commonly caused by somatic variants in the c-Kit gene or the platelet-derived growth factor receptor alpha (PDGFRA) gene. In contrast, wild-type GISTs lacking these somatic drivers can harbour SDHA variants in up to 50% of cases (Boikos *et al.* (5)). In such circumstances, a germline SDHA variant is more likely to be contributory and would typically be considered actionable, warranting surveillance for PPGLs and cascade testing, as in case 2.

However, if a somatic c-Kit or PDGFRA driver mutation is identified, these events are usually mutually exclusive with SDH deficiency, making it less likely that the GIST is attributable to the germline SDHA variant. In case 1, the presence of a PDGFRA driver together with preserved SDHA immunohistochemistry suggested that the tumour was not SDHA-deficient. Although truncating SDHA variants (such as the one identified here) have been reported in SDH-deficient GIST, in the absence of IHC loss or SDH-related pathology, this variant was regarded as non-actionable in the UK practice context.

That said, it is important to acknowledge the limitations of IHC in this setting. Where a pathogenic SDHX variant is identified, especially with supporting evidence such as loss of heterozygosity (LOH) and no alternative oncogenic driver, the overwhelming likelihood is that SDH expression would be lost, and IHC contributes little additional clinical value. In this context, preservation of IHC expression would not alter the interpretation of a clearly pathogenic variant. IHC is most useful in the absence of molecular data, rather than as confirmatory evidence when molecular findings are already present. A useful analogy can be drawn from BRCA1/2 variants in prostate or pancreatic cancer, where pathogenicity is assumed without requiring homologous recombination deficiency testing to ‘prove’ biological relevance. In contrast, for cancers with multiple well-described oncogenic pathways (e.g. colorectal cancer), IHC or additional functional testing may be more critical, since not all tumours arise through the same mechanism. By comparison, the driver spectrum in GIST is more constrained, and fewer hits are required, so the role of IHC is less decisive.

The complexity is further illustrated when an SDHA variant is identified in an individual without a GIST or PPGL. In such cases, UK guidance (7) recommends the use of SDHA immunohistochemistry on tumour tissue, where available, to clarify whether the tumour is SDHA-related. In case 3, immunohistochemistry showed preserved SDHA and SDHB staining in a prostate cancer specimen, consistent with the tumour being unrelated to the germline variant. Based on current UK guidance, this was considered non-actionable. Nonetheless, we recognise that, given the reported penetrance of SDHA variants and examples of SDH-deficient GISTs arising without family history (8), other healthcare systems may recommend surveillance for the proband and/or relatives.

**Figure 1:**
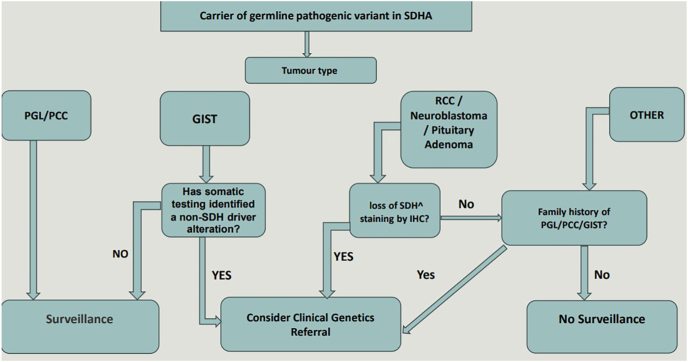
A structured clinical algorithm summarising the interpretation and management of SDHA variants. This flowchart integrates tumour phenotype, SDH immunohistochemistry (IHC) and germline findings to guide whether an SDHA variant should be considered ‘actionable’. In this context, an actionable SDHA variant refers to a germline or tumour finding that warrants clinical follow-up, including surveillance for SDH-related tumours and/or cascade testing for at-risk relatives. The algorithm begins with the identification of an SDHA-mutated tumour (e.g. GIST, phaeochromocytoma/paraganglioma or less typical SDH-associated tumours – such as RCC, neuroblastoma or pituitary adenoma). Subsequent steps assess the following: i) whether somatic sequencing identifies a non-SDH driver mutation, which may reduce the likelihood that the SDHA variant is pathogenic or clinically relevant; ii) whether SDHA immunohistochemistry demonstrates SDH-deficient staining, supporting a pathogenic role for the variant; and iii) whether there is a family history of SDH-associated tumours, which strengthens the case for an inherited predisposition. Taken together, these features help determine whether the SDHA variant is likely to represent true SDH deficiency with implications for the patient and family (‘actionable’), or whether routine surveillance and genetic referral are not indicated. The algorithm reflects current UK guidance but acknowledges that clinical practice varies internationally.

A structured approach to interpretation, such as the algorithm in Fig. 1, can support clinicians in deciding when an SDHA variant requires further action (e.g. tumour surveillance or family cascade testing). This framework highlights the importance of integrating tumour phenotype, IHC and germline results, while also acknowledging variation in international practice. Such an approach allows for tailored care, ensuring that interventions are targeted to those most likely to benefit, while minimising unnecessary surveillance and the associated psychological and logistical burden.

## Declaration of interest

Professor Rosalind Eeles has the following conflicts of interest to declare: Honoraria from GU-ASCO, Janssen, University of Chicago and Dana Farber Cancer Institute, USA, as a speaker; educational honorarium from Bayer and Ipsen; a member of external expert committee for AstraZeneca UK; and a Member of Active Surveillance Movember Committee. She is a member of the SAB of Our Future Health. She undertakes private practice as a sole trader at The Royal Marsden NHS Foundation Trust and 90 Sloane Street SW1X 9PQ and 280 Kings Road SW3 4NX, London, UK. Other authors have no conflicts of interest to declare.

## Funding

We acknowledge support from the NIHR to the Biomedical Research Centre at The Royal Marsden NHS Foundation Trust and The Institute of Cancer Research.

## Author contribution statement

E Mohamed is the first author and contributed to case 2, including the clinical assessment, data collection and drafting of the manuscript. Dr Morganstein and Dr McViegh contributed to case 1, including patient management and clinical input. Prof. Eeles contributed to case 3 and provided expert oversight and input throughout the writing process. All authors have reviewed and approved the final version of the manuscript. Permission has been obtained from the responsible physicians for the inclusion of each case.

## Patient consent

Written informed consent has been obtained from the patients for publication of the submitted article and accompanying images. A signed copy of the consent form has been retained in the patient’s medical records, in accordance with the journal’s patient consent and confidentiality policy.

## Patient’s perspective

Case 1: The patient acknowledges the concerns regarding cost-effective screening for individuals with secondary SDHA mutations but emphasises the differing perspectives among patients. While some may feel burdened by additional testing, others would find reassurance in periodic monitoring. The UK’s approach of not recommending surveillance contrasts with international practices, where regular blood pressure checks, metadrenaline tests and imaging every 3–5 years are considered. In addition, genetic testing for first-degree relatives could provide valuable insight for future health monitoring and family planning. The patient questions whether an ‘all or nothing’ approach is ideal, suggesting that cost-effective measures, such as self-arranged annual metadrenaline tests, could be a reasonable middle ground. The evolving understanding of SDHA mutations raises concerns about how patients who are not monitored today might be informed of future medical advancements that could impact their care. Clear and empathetic communication is required to reassure patients when testing is not indicated.
